# Patient-Led Smartwatch ECG Follow-Up Strategy After AF Ablation: Clinical Trial Design and Implementation

**DOI:** 10.1016/j.jacadv.2025.102534

**Published:** 2026-01-28

**Authors:** Nikhil Ahluwalia, Hakam Abbass, Ahmed Hussain, Gunkavee Saengkrajang, Rangeena Assadi, Charles Butcher, Edd Maclean, Michele Orini, Malcolm Finlay, Shohreh Honarbakhsh, Ross J. Hunter, Richard J. Schilling

**Affiliations:** a Electrophysiology Department, Barts Heart Centre, St Bartholomew’s Hospital, London, United Kingdom; b William Harvey Research Institute, Queen Mary University of London, London, United Kingdom; c Department of Biomedical Engineering, King’s College London, London, United Kingdom

**Keywords:** atrial fibrillation, catheter ablation, digital health, electrocardiography, smartwatch, wearable devices

## Abstract

**Background:**

Conventional follow-up after atrial fibrillation (AF) catheter ablation relies on physician-led interval monitoring and often fails to characterize paroxysmal symptoms. An increasing number of patients use smartwatch-based ECG devices for rhythm monitoring, but their structured integration into clinical workflows and the handling of the resultant data are not well described.

**Objectives:**

To describe the design, operationalization, data pipeline, and user engagement of a patient-led smartwatch ECG follow-up strategy after AF ablation within a randomized clinical trial.

**Methods:**

A prospective, randomized controlled trial of adults undergoing first-time AF ablation was conducted. Participants were randomized to an Apple Watch-based protocol (daily and symptom-triggered ECGs) or standard follow-up. A prespecified audit of the smartwatch-derived rhythm classification was conducted. User engagement, symptom annotation, and downstream resource use were quantified. Primary clinical outcomes are reported in a companion Brief Report.

**Results:**

Of the 168 enrolled participants (mean age 60.5 ± 9.9 years, 52 (31.0%) female, 84 (50.0%) persistent AF), Active-arm participants recorded a median of 170 (IQR 93–380) ECGs over 12 months and transmitted a median of 1.9% (0.0–8.3) for review. Symptom-annotated ECGs were more likely to show AF compared with unannotated ECGs (OR 16.1, 95% CI 13.0–19.9, *P* < 0.001) Watch-derived AF and sinus rhythm labels had positive predictive values of 0.96 and 0.95 respectively, although one-third of ECGs were unclassified.

**Conclusions:**

A structured, patient-led smartwatch ECG workflow can be embedded into routine post-ablation care with high engagement, modest staff workload, and accurate device-level rhythm classification. This implementation framework provides a practical template for integrating patient-generated wearable data into AF follow-up pathways and future digitally enabled trials.

## Graphical Abstract

**Figure:**
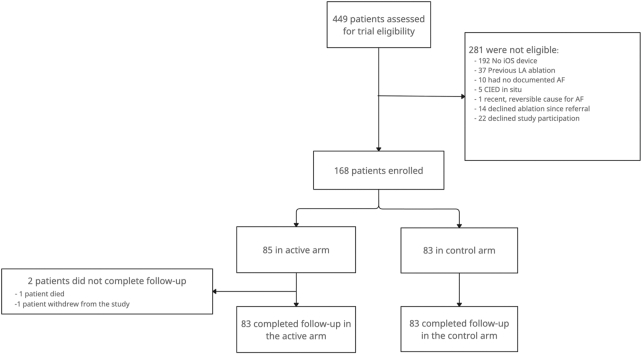

Atrial fibrillation (AF) is the most common chronic arrhythmia, with a lifetime risk of 1 in 3 to 5 in adults over 45 years.^1^ The principal direct cost associated with AF in the United States is hospitalization-related, with a median annual cost of $40,717 per patient.^2^ The direct annual costs of AF to the UK National Health Service (NHS) are £2.2 billion, also driven by unplanned hospital admissions.^3^ Catheter ablation (CA) is the most effective rhythm-control therapy for improving AF symptoms and heart function.^4^^,^^5^ However, symptoms can recur in up to 50% of patients, and these can be challenging to characterize when transient.^6^^,^^7^ Nevertheless, characterization is a critical step; adjudication of triggered activations in patients who received continuous rhythm monitoring after CA demonstrated only 45% correlated with an atrial arrhythmia.^8^ AF recurrence occurs in 30% to 50% of patients receiving conventional CA treatment, and reducing time-to-AF recurrence detection may enable earlier treatment decision-making, reducing time spent in AF.

Patient-owned wearable devices, such as the Apple Watch (AW), offer high-frequency photoplethysmography (PPG)-based heart rate and rhythm monitoring, with an on-demand, clinical-grade single-lead electrocardiogram (ECG) recording facility.^9^^,^^10^ Despite widespread adoption, there is limited evidence describing their operationalization within structured clinical workflows and how the resultant data should be managed.

This randomized controlled trial (RCT) was designed to assess whether a patient-led, watch-based monitoring strategy could be integrated into routine postablation workflows and applied at scale using commercially available consumer technology. The primary clinical outcomes are reported in the accompanying report in *JACC*.^9^ In this article, we provide a detailed description of the trial design and operationalization frameworks with practical considerations for implementing patient-led wearable data into clinical pathways.

## Methods

### Study population

This prospective study screened consecutive patients with paroxysmal or persistent AF who were referred by an electrophysiologist for first-time CA for AF to the study site. A participant was defined as having paroxysmal AF if all recorded episodes terminated in under 7 days, spontaneously or with electrical or chemical cardioversion.^5^ Persistent AF was defined as sustained AF for more than 7 days.

Patients with any recent, reversible cause for AF or continuous AF for >3 years were not eligible. Adults (21 years old or older, with no upper age limit) with access to an iPhone running the latest operating system who provided informed consent were enrolled before their CA and subsequently randomized to either the control or active arm (full enrollment criteria in Supplemental Table 1). Patients with paroxysmal AF symptoms following an initial diagnosis but without a recent ECG recording of AF within 12 months of the screening date were excluded.

### Study design

This investigator-initiated, single-center RCT was designed, implemented, and reported in accordance with the International Conference on Harmonisation Good Clinical Practice Guideline.^10^ Study data were collected and managed using REDCap electronic data capture tools hosted at Queen Mary University of London.^11^

The study was prospectively registered on a public database (NCT05016791) and received approval from the NHS Research Ethics Committee (21/WM/0228). The results of the study are reported by the Consolidated Standards of Reporting Trials checklist for RCTs (Supplemental Table 2). The study design and patient protocol were developed with input from patient representatives at Arrhythmia Alliance UK, who will also support the dissemination of findings through their network.

### Randomization and study intervention

Unblinded, randomized allocation was performed before CA in a 1:1 ratio in blocks of 4, to either the active or control study arm, and stratified by AF type (Redcap Randomization module, Redcap).

Participants in the active arm were loaned a commercially available AW Series 5 (Apple Inc) and prescribed a high-frequency wearable-based monitoring protocol described below. Participants in the active study arm who owned AW (Series 5 or later) with the ECG app were permitted to use their own device.

AW periodically collects heart rate data using a wrist-facing PPG sensor. Heart rate measurements are taken at least 15 minutes apart when no specific workout is active. A proprietary algorithm analyzes the PPG trace to detect episodes of irregular heart rate, suggesting possible AF.^12^ If 5 out of 6 sequential readings within 48 hours suggest possible AF, the watch displays a push notification of an irregular rhythm alert and to consider recording an ECG. An ECG, comparable to lead I of Einthoven’s triangle, can then be recorded using 2 integrated electrodes on the back and the digital crown. ECG recording can also be done at any time initiated by the user, and symptoms can be temporally annotated to the ECG. ECG data are stored on the patient’s smartphone.

As part of the study protocol, participants were asked to perform daily ECGs until their 12-month follow-up date or when they became symptomatic or were prompted to do so by the PPG-based AF detection algorithm on their AW when worn. They received guidance on how to record an ECG.

Standard care for the control arm consisted of interval clinical appointments at 3, 6, and 12 months, during which an AF specialist health care professional evaluated the ECG assessment and clinical status. Ad hoc, symptom-based monitoring was also performed with 12-lead ECG or Holter monitoring of variable duration conducted by the evaluating clinician. This practice is standard care at the study institution and in line with the pattern and intensity of postablation monitoring for clinical care suggested by contemporary international expert consensus.^13^

### Catheter ablation

All CA procedures were performed on uninterrupted anticoagulation, commenced at least 1 month prior; otherwise, preprocedural transoesophageal echocardiography was used to rule out left atrial appendage thrombus. Procedures were performed under local anesthesia and conscious sedation, unless there was a coexisting indication or preference for general anesthesia. The ablation technology and lesion set were at the discretion of the clinical operator, with pulmonary vein isolation as a minimum requirement.

### Follow-up

The date of the index CA was defined as day 0 for follow-up. The clock was not reset if repeat CA was undertaken. Participants who did not undergo CA after enrollment remained in the study to mitigate exclusion bias that may be related to group allocation, and their date of enrollment was defined as day 0. Day 0 to 90 after CA was defined as the blanking period in accordance with international consensus and guidelines.^5^^,^^13^ AF events detected during this period were recorded but not counted toward the primary endpoint, in line with contemporary trials and clinical practice.^14^ Participants were followed up at 3, 6, and 12 months after CA, with a routine ECG performed at each visit. Quality of Life (QOL) was assessed using the AF Effect on Quality-of-Life questionnaire (AFEQT) score (range: 0-100 from worst to best QOL) at 6 and 12 months, with the latter used to determine change in score after CA. Class 1c or class III antiarrhythmic drugs (AADs) were routinely discontinued at 3 months in participants with sinus rhythm. Oral anticoagulation was continued beyond the 3-month time point based on stroke risk stratification scoring and shared decision-making. Prespecified harms, including procedure-related adverse events, were systematically captured at follow-up appointments. To mitigate any misdirection of clinical concerns to the research team, participants provided verbal and written confirmation of understanding that the study did not replace their routine clinical care.

AF recurrence was defined as any documented sustained atrial arrhythmia on a 12-lead ECG or >30 seconds on a single-lead ECG technology.^5^ Participants in the active arm were able to transmit their smartwatch ECGs for overreading and classification only to a secure NHS.net mailbox with access limited to selected members of the research team. The responsible research team consisted of 3 health care professionals (N.A., H.A., R.J.S.), of whom 2 were accredited to interpret ECGs clinically. ECGs received outside of standard working hours were reported in the following working period, with an expected period of <72 hours to review transmitted data. Participants confirmed at the time of consent that they were aware that ECG transmission was for research study purposes and that they should seek independent medical review as appropriate. If the smartwatch ECG showed a first AF recurrence outside the blanking period, the research team forwarded it to the patient’s treating cardiologist.

Participants in the control group were instructed to notify the clinical arrhythmia service (a nurse-led service available at the study site during working hours) or their treating physician if they experienced any symptoms suggestive of arrhythmia. A subsequent ECG or Holter monitor was ordered based on the clinician's suspicion and symptom frequency. Commencement of AADs or repeat CA was at the discretion of the referring clinician.

### Data handling

Upon completing the study, participants in the active arm exported their Health data from their iPhone in .xml format via the Health app directly to the research team. All ECG recordings, heart rate data, wear time, and activity data were downloaded and parsed using a bespoke Python workflow adapted from an open-source HealthKit XML parser.^15^ This was internally validated on known HealthKit data sets to ensure reproducible extraction and stable performance over time.

The pipeline extracted all ECG recordings as a raw waveform in .csv format. Associated metadata, including timestamps, ECG app-derived rhythm classification labels, and user-annotated symptom labels, were converted into time-ordered dataframes per participant using a structured master script. Timestamps were standardized to device-recorded local time. ECG-timestamped data were used for analytic endpoints; no interpolation or imputation of missing data was performed.

### ECG classification

The ECG app (AW Series 4 and later) uses proprietary algorithms for PPG-based AF detection and ECG rhythm classification.^12^ The app classifies ECG recordings as showing AF or as showing sinus rhythm with no signs of AF, and these were considered for the analytical endpoints. Equivocal ECGs are not classified and labeled as inconclusive. ECGs labeled by AW as “high heart rate,” “low heart rate,” or “poor recording” were also considered as not classified.

The accuracy of the ECG classification feature on this smartwatch has not been reported in patients with chronic arrhythmia. Therefore, a pilot accuracy analysis was planned after study participants transmitted the first 1,000 ECGs to the research team. The ECG recording was deidentified and declassified before being reviewed by 2 cardiac electrophysiologists for gold-standard classification. Agreement analysis was conducted independently to assess the accuracy of the ECG classification feature in this patient cohort. To assess for potential information missingness related to user-driven transmission behavior based on the label, we quantified the distribution of smartwatch-applied classification labels across all recorded ECGs and compared this with the label distribution within the transmitted subset.

### Sample size

The trial was powered for the primary endpoint based on comparable studies reporting continuous monitoring event rates after CA.^7^^,^^14^ We assumed a 12-month recurrence detection rate of 48% in the active arm vs 26% in the control arm. Using a log-rank test (2-sided α = 0.05, 80% power) and allowing for 10% loss to follow-up, the required sample size was 168 participants.

### Statistics

The Shapiro-Wilk test was used to determine whether the data were normally distributed. Continuous variables were analyzed using a 2-tailed independent *t*-test for normally distributed data or the Mann-Whitney *U* test for non-normally distributed data. The chi-squared test was used for categorical variables. Normally distributed data were presented as mean ± SD, and non-normally distributed data as median (lower-upper quartile). The significance level for all tests was set at *P* < 0.05. Deidentified participant data, statistical code, and related materials are available from the corresponding author on reasonable written request.

The primary endpoint reported in the companion *JACC* paper was analyzed using a Cox proportional hazards model.^9^ To account for the 90-day blanking period landmark sensitivity analysis in which time-zero was redefined at day 90 postablation was performed. This analysis was used to evaluate the robustness of the primary Cox model to the choice of time origin and to assess potential immortal-time bias. Proportional hazards assumptions were tested using Schoenfeld residuals. For time-to-event analyses, participants without a documented recurrence were right-censored at their last follow-up. An intention-to-treat analysis was performed according to their randomized allocation, preserving participants in the control arm who used their own smartwatch for self-monitoring in their allocated group. Sensitivity analyses were conducted to include events in the blanking period and to cross over participants in the control arm who owned AWs to the active arm. The rate of unplanned hospitalization was analyzed both as a binary outcome using chi-square testing and using negative binomial regression to estimate incidence rate ratios. For secondary binary and count outcomes, analyses were conducted on an available-case basis and no imputation for missing data was performed.

## Results

### Participant characteristics

**Figure 1:**
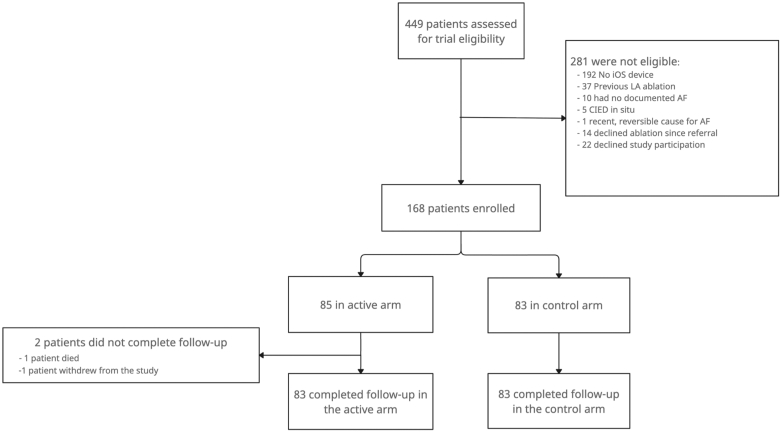
**CONSORT Flow Diagram Showing Participant Progression Through Trial Stages, With Numbers and Reasons for Exclusion** AF = atrial fibrillation; CIED = cardiac implantable electronic device; CONSORT = consolidated standards of reporting trials; iOS = iPhone operating system; LA = left atrium.

A total of 449 patients were assessed for eligibility between January 2022 and January 2024. Of these, 168 participants were enrolled and randomized. A total of 281 (62.6%) did not meet the inclusion criteria (Figure 1). Of the screened participants, 192/449 (42.8%) were excluded because they did not have an iPhone. In an initial survey, 95% of these patients owned a smartphone from a different manufacturer.^16^ The mean age of enrolled participants was 60.5 ± 9.9 years; 52 (31.0%) were female, and 84 (50.0%) had persistent AF. There were no significant differences in demographics, comorbidities, or AF type between participants screened but not enrolled and those enrolled (Supplemental Table 3).

There were no significant differences in demographics or comorbidity prevalence between the active and control study arms. Baseline characteristics are presented in Table 1. Twenty-one (25.3%) participants in the control arm reported owning and using an AW device.

**Table 1:** Baseline Characteristics of Study Participants by Arm

	Control (n = 83)	Active (n = 85)	*P* Value
Age (y)	59.5 ± 9.9	61.2 ± 9.8	0.271
Female	24 (28.9)	24 (28.2)	1.000
Body mass index (kg/m^2^)	28.3 ± 4.2	28.9 ± 5.0	0.646
Ethnicity			0.565
White	76 (91.6)	79 (92.9)	
Asian or British Asian	4 (4.8)	4 (4.7)	
Black, African, Caribbean, or British Black	3 (3.6)	1 (1.2)	
Mixed or multiple ethnic groups	0	1 (1.2)	
Other	0	0	
Hypertension	28 (33.7)	35 (41.2)	0.403
Hypercholesterolemia	17 (20.5)	18 (21.2)	1.000
Ischemic heart disease	8 (9.8)	16 (18.8)	0.147
LVEF categorization			0.597
Preserved	60 (73.2)	64 (76.2)	
HFmrEF	9 (11.0)	11 (13.1)	
HFrEF	13 (15.9)	9 (10.7)	
Diabetes mellitus	9 (10.8)	6 (7.1)	0.556
COPD or asthma	8 (9.6)	15 (17.6)	0.200
Obstructive sleep apnea	1 (1.2)	4 (4.7)	0.378
Thyroid disease	9 (10.8)	7 (8.2)	0.754
Stroke or TIA	5 (6.1)	7 (8.2)	0.797
CHADS_2_VASC score	1.5 ± 1.3	1.6 ± 1.3	0.481
Alcohol consumption			0.922
Nil	25 (30.1)	24 (29.6)	
Less than RWA	54 (65.1)	52 (64.2)	
Greater than RWA	4 (4.8)	5 (6.2)	
AF type			0.916
Paroxysmal	42 (50.6)	42 (49.4)	
Early persistent	32 (38.6)	32 (37.6)	
Long-standing persistent	9 (10.8)	11 (12.9)	
Persistent AF duration (mo)	11 (7, 19)	10 (6, 13)	0.303
Previous DCCV	44 (54.3)	37 (45.7)	0.200
Modified EHRA class			0.855
1	2 (2.4)	1 (1.2)	
2	40 (48.7)	43 (51.2)	
3	38 (46.3)	39 (46.4)	
4	2 (2.4)	1 (1.2)	
AFEQT score	57 ± 21	58 ± 24	0.803
Medications			
Beta-blocker	65 (78.3)	64 (75.3)	0.779
Calcium-channel blocker (nondihydropyridine)	2 (2.4)	3 (3.6)	1.000
Digoxin	9 (10.8)	6 (7.1)	0.556
Any antiarrhythmic drug	33 (39.8)	36 (42.4)	0.853
Any renin-angiotensin-aldosterone system inhibitor	31 (37.3)	35 (41.2)	0.846
Direct oral anticoagulation	68 (81.9)	74 (87.1)	0.48
Warfarin	1 (1.2)	2 (2.4)	1.000

Values are mean ± SD, median (IQR), or n (%).

AF = atrial fibrillation; AFEQT = atrial fibrillation effect on quality-of-life questionnaire (range: 0-100); COPD = chronic obstructive pulmonary disease; DCCV = direct current cardioversion; EHRA = European heart rhythm association; HFmrEF = heart failure with mildly reduced ejection fraction; HFrEF = heart failure with reduced ejection fraction; LVEF = left ventricular ejection fraction; RWA = recommended weekly alcohol allowance (<14 U); TIA = transient ischemic attack.

### AF ablation characteristics

A total of 166 participants underwent CA. Two (1.1%) did not, due to their decision on the day of the procedure, attributed to an interval change in their symptom burden. There was no significant difference in index ablation technology or strategy between the 2 arms. Procedural characteristics are reported in Table 2. Repeat CA was performed in 38 participants (22.6%). Two (1.0%) procedure-related adverse events occurred, both adjudicated unrelated to study allocation. One patient had an asymptomatic phrenic nerve palsy following cryoablation, and the second had a pericardial effusion requiring a pericardial drain that was in situ for 12 hours. The patient was discharged home the following day.

**Table 2:** Ablation Procedure Characteristics by Study Arm

	Control	Active	*P* Value
Sedation (non-GA)	70 (85.4)	67 (79.8)	0.456
Ablation type			0.672
Cryoballoon	42 (51.9)	47 (56.6)	
Radiofrequency point-by-point	38 (46.9)	35 (42.2)	
Other	1 (1.2)	1 (1.2)	
PVI	80 (98.8)	82 (98.9)	1.00
Extra-PVI ablation	15	18	0.610
Complications	0	2	0.510^a^
Procedure duration (min)	112.7 ± 64.2	107.5 ± 60.7	0.616

Values are mean ± SD.

GA = general anesthetic; PVI = pulmonary vein isolation.

a Fisher exact test used due to low expected counts.

### Follow-up

Two participants in the active arm did not complete follow-up; one due to relocation, and a second died during the study period due to an unrelated cause (hepatobiliary cancer). All other participants completed 12 months of follow-up after the index CA.

At 3 months follow-up, AAD use (40 [47.1%] vs 37 [44.6%]; *P* = 0.867) and β-blocker use (59 [69.4%] vs 65 [78.3%]; *P* = 0.256) were similar in the active and control arms. AAD use was reduced over the follow-up period in both groups, with a similar proportion of patients taking these medications at 12-month follow-up. (14 [16.9%] vs 15 [18.3%]; *P* = 0.971). Fewer Holter monitors were conducted in participants in the active arm (incidence rate ratio: 0.51 (95% CI: 0.28-0.96); *P* = 0.035). Thirty-eight Holters were performed in 28 (33.7%) participants in the control arm, and 20 Holters were performed in 18 (21.7%) participants in the active arm during their 12-month follow-up. No arrhythmia was detected in 19/28 (67.9%) patients in the control arm, who were in sinus rhythm throughout their test.

AFEQT scores improved in both arms of the study after CA (56 ± 23 to 76 ± 24; *P* < 0.001). There was no difference in the final follow-up score between the 2 groups (78 ± 23 vs 76 ± 23; *P* = 0.754). Participants with AF recurrence had a lower AFEQT score at follow-up (62 ± 25 vs 89 ± 14; *P* < 0.001), seen in both the active (67 ± 26 vs 89 ± 13; *P* < 0.001) and control (56 ± 22 vs 89 ± 14; *P* < 0.001).

### Participant engagement

**Figure 2:**
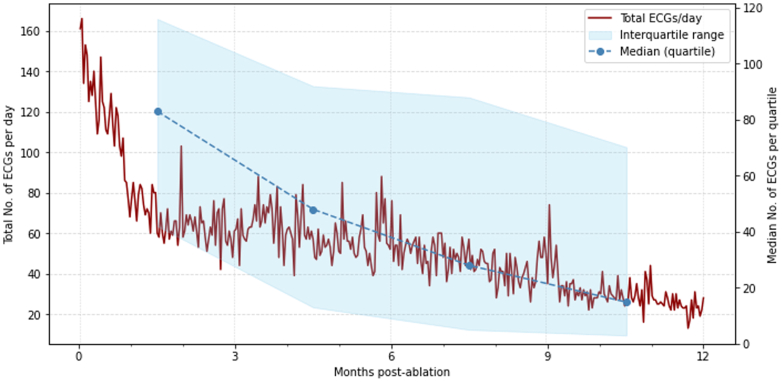
**Line Charts to Show ECG Recording Activity in the Active Arm During 12-Month Follow-Up** The total number of ECGs recorded per day (red line) across all participants and the median number of ECGs recorded per quartile (blue points) with a shaded area to indicate 95% CIs are shown ECG = electrocardiogram.

The frequency of ECG recordings was highest in the first quartile of follow-up and then declined across subsequent quartiles (*P* < 0.001) (Figure 2). Thirty-eight (44.7%) participants recorded ECGs every month of their follow-up period. Participants in the active arm with an AF recurrence during follow-up recorded more ECGs than those without (269 [134-519] vs 188 [57-280]; *P* < 0.001).

A total of 825 (4.1%) ECGs were contemporaneously annotated with symptoms. Seventy-six (89.4%) participants in the active arm annotated at least 1 ECG with symptoms. The most commonly annotated symptom was rapid heart rate, observed in 677 (82.1%) ECGs. Of those classified, symptom-annotated ECGs were more likely to be classified as AF compared with unannotated ECGs (433 [79.6%] vs 3,282 [19.5%], OR: 16.1; 95% CI: 13.0-19.9; *P* < 0.001). A total of 111 (13.5%) symptom-annotated ECGs were classified as sinus rhythm, and 281 (34.1%) were unclassified.

During follow-up, unclassified ECGs were accompanied by a repeat ECG recording on the same day in 2,371 (88.6%) cases. The repeat, same-day ECG was classified as either sinus rhythm or AF on 1072 (69.2%) occasions.

A median of 1.9% (0.0-8.3) of recorded ECGs were transmitted, with 24 (28.2%) participants transmitting none of their recorded ECGs for rhythm characterization. The number of ECGs transmitted was inversely correlated with their AFEQT score at follow-up (r = −0.370; *P* = 0.005).

### ECG classification feature evaluation

**Figure 3:**
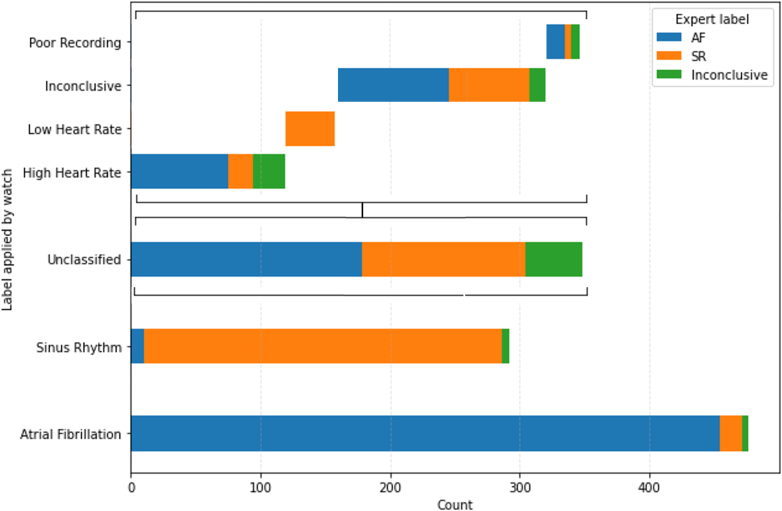
**Stacked Bar Chart Showing Smartwatch-Assigned Rhythm Labels and Corresponding Expert Over-read Classifications** Unclassified subtypes are displayed in a waterfall format to illustrate component breakdown. SR = sinus rhythm; other abbreviation as in Figure 1.

The accuracy of the ECG classification feature was evaluated using the first 1,093 consecutive AW ECGs transmitted. A median of 6 (2-17) ECGs were included from the initial 52 active-arm participants. ECG transmission was label-dependent, with recordings labeled as AF, high-rate, or inconclusive more frequently transmitted than those labeled by the watch as sinus rhythm as a proportion of the number recorded (*P* < 0.001). Of these, 475 (43.5%) were classified as AF by the AW. Compared to blinded expert adjudication, AF classification by the smartwatch had a positive predictive value of 0.96 and a specificity of 0.96. A total of 276 (25.2%) ECGs were classified as sinus rhythm by the smartwatch with a positive predictive value of 0.95 and a specificity of 0.98 (Figure 3). A total of 342 (31.3%) ECGs were not classified as either sinus rhythm or AF by the smartwatch. Most unclassified ECGs were labeled as “inconclusive” (n = 161, 14.7%) or “high heart rate” (n = 119, 10.9%). Of the unclassified ECGs labeled as “high heart rate,” 68 (57.1%) were adjudicated as AF, and 7 (5.9%) showed atrial tachycardia (Table 2).

## Discussion

### Main findings

This article details the trial design, workflow, and operational experience underpinning this first RCT of patient-led, smartwatch-based ECG monitoring in an established clinical pathway. The feasibility of implementing a structured protocol using a commercially available ECG-recording device for clinical care is demonstrated, and the accuracy of the classification algorithm in a high-risk clinical cohort is reported. The clinical utility of this intervention is presented in the companion *JACC* report.^9^

### Device performance

The Apple Heart Study demonstrated the accuracy of the device’s PPG-based screening algorithm in a healthy self-selected population that owned AW Series 1 to 3 devices.^17^ Internal validation studies of the ECG classification feature demonstrated a specificity of 99.3% and a sensitivity of 98.5%. It has received both Food and Drug Administration clearance and a Conformité Européenne (CE) mark.^12^ However, limited external evaluation has been conducted in clinical populations. A small study of 50 patients after cardiac surgery reported a sensitivity of 96% and a specificity of 100% compared with telemetry; however, it was conducted in a controlled environment and excluded patients with chronic arrhythmias.^18^ In contrast, our analysis reports the performance of this feature in an ambulatory cohort with a high pretest probability of AF, as well as other arrhythmias and ectopy.^19^ Our enrollment criteria were chosen to reflect the cohort commonly encountered in clinical practice, with CHADS2VASc risk factors, and in whom symptom discrimination and the detection of AF impacts treatment decisions. Although we recommended a monitoring protocol, we chose not to implement interval behavioral nudges or conduct compliance evaluations. This was to allow pragmatic use of a commercially available product in a scalable, low-workload methodology. The per-treatment sensitivity analysis showed that AW ownership alone in the control arm did not reproduce the treatment effect, suggesting that the structured monitoring protocol may be integral to higher likelihood of AF detection.^20^ The AW ECG classification feature was utilized for patient-level triage of their recorded ECGs to facilitate the broader application of the protocol and reduce manual review. Expert reading reclassified 52% of 342 unclassified ECGs as AF. However, the majority of participants repeated their ECG recording after receiving an unclassified label, demonstrating self-directed attempts at clarification that would reduce this workload burden during pragmatic implementation.

### Clinical service integration

Current guidelines and international consensus support symptom-guided follow-up after CA and recommend ECG documentation when symptoms raise diagnostic uncertainty.^5^^,^^21^ Active arm participants also annotated ECGs showing sinus rhythm with symptoms, demonstrating the need for real-time ECG characterization. Some centers implement noninvasive monitoring at increased intensity. However, this reactive model of physician-ordered investigations can still delay the characterization of paroxysmal symptoms if they do not coincide with their occurrence.^22^ Augmentation with patient-led PPG-based rhythm monitoring after CA can improve event detection compared to symptom-guided rhythm monitoring alone and provide an estimate of AF burden.^23^^,^^24^ However, it has limited sensitivity due to indeterminate results and is insufficient for diagnosis; patients still require subsequent ECG characterization. Patient-led monitoring using a portable ECG device has demonstrated feasibility in detecting AF recurrences after ablation without additional ECG characterization.^25^ Our study implemented an RCT design to evaluate the relative impact on arrhythmia-related and clinical endpoints over a clinically relevant follow-up duration.

### Operationalization considerations

Most participants transmitted a very small proportion of their recorded ECGs, usually in the context of AF-related symptoms and AF recurrence, suggesting appropriate use. Although research participation did not substitute for clinical care in this study, fewer clinical Holter monitors were requested for participants in the active arm. The review time for ECG transmissions was short; accepting this may reflect the limited research remit, smartwatch-derived ECGs may be justifiably integrated into existing workflows if the net workload burden of managing the postablation patient cohort is reduced.

The backend data-handling pipeline was developed using bespoke scripts that could be adapted to harmonize ECG data and metadata from different devices. An app-based data pipeline would streamline data transmission and enable the use of manufacturer-agnostic rhythm characterization algorithms with greater sensitivity, accepting a potentially higher false positive rate for this clinical cohort. Such algorithms could be specifically trained to better distinguish common causes of nonclassification, such as frequent ectopy and very short, nondiagnostic AF episodes. A physician-facing dashboard layered on top of this pipeline would further support scalable integration of these high-volume data streams into clinical workflows or, alternatively, offload the follow-up burden to an outsourced virtual monitoring service provider.

Participants with a recorded AF recurrence transmitted more ECGs. One explanation for the lower rate of unplanned hospitalization reported in the active arm may be reassurance provided by real-time symptom classification, which would otherwise prompt medical attention for characterization.^9^ Prior retrospective work has linked consumer-initiated wearable use in AF to heightened anxiety and symptom preoccupation.^26^ Participants in our trial were prospectively enrolled and provided an AW specifically as a research tool following CA, rather than self-initiating device use in response to symptoms. This framing may mitigate any behavioral factors, as QOL improved similarly across both arms. The broader impact of wearable-based monitoring on health care utilization and behaviors in older adults without known AF will be reported in the ongoing Heartline study.^27^

### Governance and data privacy considerations

A patient-led, clinical-grade monitoring protocol can empower patients to take an active role in their care. It can also facilitate care in the community through digitally enabled self-management and virtual follow-up models. However, the resultant patient-generated health data present challenges relating to data ownership, medicolegal responsibility, and compliance with privacy frameworks. The locus of accountability for responding to device-detected arrhythmias remains undefined. Clear governance is required to determine whether alerts should trigger primary-care review, specialist triage, or automated pathways, with implications for liability and resource planning.

Our findings suggest that data from approved consumer-grade wearables can be integrated into postablation care pathways as a scalable, low-resource adjunct to traditional monitoring. However, broader clinical adoption will require dedicated, secure transmission channels and interoperability with the Fast Healthcare Interoperability Resources. Once established, this infrastructure could support community-based care at scale and enable the use of wearable-derived data across other clinical pathways where high frequency physiological monitoring may provide clinical value.

### Study Limitations

Participation required access to a compatible iPhone and the ability to operate the AW, potentially introducing selection bias toward a more technologically literate population and limiting generalizability. However, participant characteristics were similar to those screened but not enrolled, and the prerequisite digital literacy required was low. Other CE-marked smartwatch ECG devices or overlaid applications may broaden accessibility, though comparative evaluations are needed.

Although the device does not provide continuous rhythm monitoring, the clinical relevance of very short episodes is uncertain.^28^ Software updates released after the study began introduced an AFib history feature to quantitatively report AF burden, which could serve as a meaningful endpoint in future research. PPG waveform data were unavailable, and symptom annotation was optional, limiting our ability to distinguish the relative yield of irregular rhythm notifications versus routine or symptom-triggered recordings. These features should be intentionally considered in the design of future app-based interventions to support smartwatch-based ECG monitoring postablation.

## Conclusions

This trial demonstrates that a structured, patient-led smartwatch ECG workflow can be operationalized and systematically evaluated within routine postablation care using commercially available technology. Future work should assess the incremental value of unified data pipelines, personalized classification algorithms with clear governance frameworks to support broader adoption of patient-generated wearable data in clinical care pathways.

Perspectives **COMPETENCY IN INTERPERSONAL AND COMMUNICATION SKILLS:** Patients may independently adopt or request patient-led rhythm monitoring using consumer-grade ECG or heart rhythm devices. Some may meet regional standards for ECG recording and AF detection. Clinicians should understand and communicate that these technologies provide intermittent, not continuous, rhythm assessment, while offering the advantage of real-time ECG documentation during symptomatic episodes. Recognizing these strengths and limitations facilitates informed, balanced discussions about their role in postablation follow-up.

**COMPETENCY IN PATIENT CARE:** Comprehensive counseling regarding the manifestations of arrhythmia recurrence, appropriate investigation pathways, and indications for medical review is essential after AF ablation. Clear instructions for symptom-triggered recordings, data transmission, and structured escalation pathways enhance patient safety and optimize the integration of wearable-derived data into routine care.

**TRANSLATIONAL OUTLOOK:** Future pragmatic studies evaluating real-world integration of smartwatch-based ECG data into clinical services are warranted to determine their impact on health care utilization and outcomes. Systematic incorporation of patient-generated ECGs into electronic health records, supported by standardized triage algorithms and clinician dashboards, may maximize clinical utility and enable scalable adoption of wearable-assisted arrhythmia care.

## Funding support and author disclosures

Prof Schilling has received research grants and educational grants from 10.13039/100000046Abbott, 10.13039/100007497Biosense Webster, and 10.13039/100004374Medtronic; has received speaker fees and travel grants from 10.13039/100000046Abbott, 10.13039/100007497Biosense Webster, and 10.13039/100004374Medtronic; and is an inventor of the STAR mapping system and is a shareholder in Rhythm AI Ltd. Prof Finlay has received research and educational grants from 10.13039/100000046Abbott and 10.13039/100004374Medtronic; has served on advisory boards for Abbott, Boston Scientific, and Medtronic; and is a shareholder and founder of Epicardio Ltd. This study was funded by a research grant from 10.13039/100015652Barts Charity and a research grant from 10.13039/100000046Abbott Inc. Apple Inc provided the Apple Watch devices for the research. Apple was not involved in the design of the research, nor was it involved in the collection, analysis, or interpretation of the research data, or the content of this or any related publication. All other authors have reported that they have no relationships relevant to the contents of this paper to disclose.
